# Síndromes Coronarianas Agudas no Contexto Atual da Pandemia COVID-19

**DOI:** 10.36660/abc.20200358

**Published:** 2020-06-29

**Authors:** Raphael Boesche Guimarães, Breno Falcão, Ricardo Alves Costa, Marcelo Antônio Cartaxo Queiroga Lopes, Roberto Vieira Botelho, Ricardo Petraco, Rogério Sarmento-Leite

**Affiliations:** 1 Instituto de Cardiologia Porto AlegreRS Brasil Instituto de Cardiologia,Porto Alegre, RS - Brasil; 2 Hospital de Messejana FortalezaCE Brasil Hospital de Messejana, Fortaleza, CE - Brasil; 3 Hospital Universitário Walter Cantídeo FortalezaCE Brasil Hospital Universitário Walter Cantídeo, Fortaleza, CE - Brasil; 4 Instituto Dante Pazzanese de Cardiologia São PauloSP Brasil Instituto Dante Pazzanese de Cardiologia,São Paulo, SP - Brasil; 5 Hospital Sírio-Libanês São PauloSP Brasil Hospital Sírio-Libanês, São Paulo, SP - Brasil; 6 Hospital Alberto Urquiza Wanderley João PessoaPB Brasil Hospital Alberto Urquiza Wanderley, João Pessoa, PB - Brasil; 7 Instituto do Coração do Triângulo UberlândiaMG Brasil Instituto do Coração do Triângulo,Uberlândia, MG - Brasil; 8 Imperial College London LondresInglaterra Reino Unido Imperial College London,Londres, Inglaterra Reino Unido; 9 Hospital Moinhos de Vento Porto Alegre RS Brasil Hospital Moinhos de Vento Porto Alegre, RS - Brasil; 10 Universidade Federal de Ciências da Saúde de Porto Alegre Porto AlegreRS Brasil Universidade Federal de Ciências da Saúde de Porto Alegre, Porto Alegre, RS - Brasil

**Keywords:** Síndrome Coronariana Aguda/complicações, Coronavirus, COVID-19, Pandemia, Infarto Agudo do Miocárdio/prevenção e controle, Telemedicina/tendências, Quarentena

## Introdução

A COVID-19, descrita inicialmente no final de 2019 na China, pode, nas formas graves, cursar como pneumonia atípica e síndrome do desconforto respiratório grave.^1^ Classificada em fevereiro de 2020 como pandemia^2^ pela Organização Mundial da Saúde (OMS), tem determinado importantes repercussões clínicas, sociais, políticas e econômicas, deixando marcas, consequências e aprendizados. A sociedade como um todo teve que se adaptar à uma nova realidade. Hospitais precisaram reescrever suas rotinas e procedimentos operacionais. A criação de cuidados especiais para evitar a disseminação interna dos vetores de contaminação tornou-se imperativa. Unidades dedicadas à COVID foram montadas, e ações protocolares de biossegurança foram instaladas. Recursos humanos, materiais e financeiros foram alocados no intuito de proporcionar a melhor qualidade assistencial possível, sem prejuízo à segurança das equipes.

O isolamento social, principal forma de conter a disseminação da doença, permitiu, em algumas localidades, o “achatamento da curva”, evitando o esgotamento total do sistema de saúde. No entanto, ainda é uma incógnita a extensão de duração da doença, risco de contágio e manutenção de todos os cuidados.

Os sintomas clássicos da COVID-19 são bem conhecidos,^3 , 4^ e a maioria dos infectados tem apresentações clínicas brandas. Todavia, em virtude das recomendações das autoridades de saúde para procurar atendimento hospitalar somente em casos graves, literalmente, do medo da população de se expor ao vírus, o diagnóstico, o tratamento e o prognóstico de várias outras condições clínicas a que usualmente os seres humanos estão expostos também têm sido duramente impactados. Isso aumenta o sinal de alerta para questões inerentes ao manejo da síndrome coronariana aguda (SCA), que pode encontrar obstáculos no atual cenário mundial.^5^

Não obstante a isso, indivíduos acima de 60 anos de idade ou que tenham doenças respiratórias, cardiovasculares prévias ou diabetes estão mais propensos a desenvolver as formas graves da COVID-19, podendo ter o seu sistema cardiovascular comprometido e sofrer manifestações de miocardite ou infartos do tipo II e fenômenos tromboembólicos.^6 , 7^

### Experiências Internacionais

Experiências internacionais de países que nos antecederam na aparição de casos apontaram associações importantes entre a COVID-19 e a doença cardiovascular.

Portadores de doença cardiovascular ou cerebrovascular acometidos por COVID-19 representam cerca de 40% dos casos graves e têm pior prognóstico.^8^ Em pacientes com COVID-19, contrastando com uma taxa de fatalidade por caso geral de 2,3%, a taxa de fatalidade por caso entre os portadores de doença cardiovascular preexistente foi de 10,5% e, entre os diabéticos, de 7,3%.^9^ Manifestações cardiológicas atribuídas à COVID-19 também foram reportadas. Arritmias ocorreram em 16,7% e lesão miocárdica aguda em 7% dos casos,^10^ com elevações de troponina registradas, particularmente nos casos mais graves.^11^

Além dessas associações diretas, “efeitos colaterais” da pandemia de COVID-19 no atendimento de SCA geraram preocupação. Registrou-se queda brusca na procura por atendimento ao pronto-socorro cardiológico pelos pacientes com SCA, possivelmente relacionada com o medo de contrair a infecção no ambiente hospitalar, que pode redundar em subdiagnóstico e tratamento inadequado, com risco de sequelas e mortes evitáveis.^12 , 13^ Além disso, atrasos para angioplastia primária foram registrados tanto pré-hospitalares, por relutância na procura ou por dificuldades de acesso, fazendo com que o paciente seja admitido em uma condição mais grave, como intra-hospitalares, atribuídos a modificações nos fluxos decorrentes das barreiras de biossegurança necessárias contra o coronavírus.^14^

Alertas à população sobre a importância de valorizar sintomas sugestivos de SCA e de procurar ajuda rapidamente são fundamentais e vêm sendo gerados por associações importantes em outros países.^15^ A telemedicina é uma ferramenta facilitadora nesse contexto, tem o potencial de permitir ao médico reconhecer remotamente sintomas suspeitos de SCA e orientar o paciente a procurar imediatamente por atendimento. Além disso, permite diagnóstico pré-hospitalar de infarto agudo do miocárdio com supradesnível de segmento ST, possibilitando acionamento rápido dos laboratórios de hemodinâmica e seleção da melhor estratégia de reperfusão miocárdica, fibrinolítica ou angioplastia primária, de maneira personalizada. Pode-se evitar a passagem pelo pronto-socorro, conduzindo o paciente diretamente à sala de cateterismo, de forma a minimizar o risco de infecção nosocomial e encurtar o tempo para recanalização, reduzindo tempo de internação e sequelas.^16 , 17^

Assim, o preparo das equipes com treinamento médico continuado, protocolos assistenciais, alertas de novas políticas públicas populacionais e o uso da telemedicina como ferramenta auxiliar têm demonstrado ser fundamentais.

### Manejo da Síndrome Coronariana Aguda *(Protocolos de Atendimento)*

A pandemia da COVID-19 fez emergir novos questionamentos, desafios e paradigmas na abordagem da SCA^18^ – uma emergência médica que deve ser diagnosticada e tratada precocemente conforme protocolos validados extensamente na literatura.^19^ É fato incontestável que o tratamento da SCA, sobretudo do infarto agudo do miocárdio, tem evoluído e demonstrado reduções significativas nas taxas de mortalidade, especialmente se implementado nas primeiras horas do evento cardiovascular.^20^ A trombólise, a angioplastia e os *stents* coronários promoveram uma verdadeira revolução. Com ações precoces, verifica-se menor número de arritmias ventriculares, redução do tamanho do dano miocárdico, menores incidências de reinfarto e maior preservação da função ventricular.^21^ Tais efeitos se sustentam a longo prazo e impactam na qualidade e expectativa de vida. Infelizmente, a pandemia da COVID-19 tem impactado negativamente no diagnóstico precoce e no adequado tratamento da SCA atualmente. São muitos os relatos apontando para uma significativa redução nos atendimentos por essa apresentação nos setores de emergências. Dados reportados de todo o mundo e da cidade de Nova York, Estados Unidos, apontam para redução de até 70% no volume de atendimentos por SCA e aumento em até 800% nas mortes súbitas.^22 , 23^

**Figura 1:**
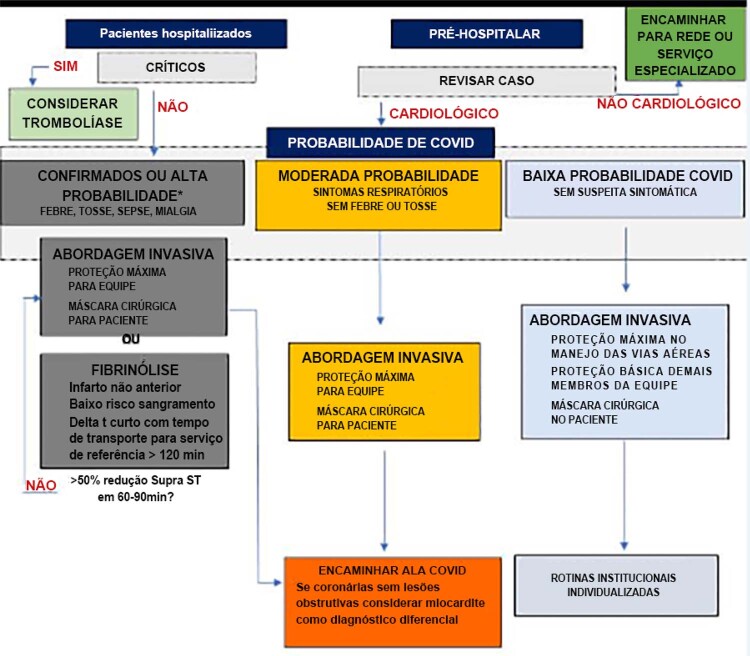
– *Infarto agudo com supra ST na era COVID.*

Recomendações de diversas Sociedades Médicas^19 , 20^ destacam as implicações clínicas cardiovasculares do coronavírus e atenção para os riscos individuais e populacionais.^20 , 21^ Além das estratégias de saúde pública para prevenção da disseminação da infecção, vacinação anti-influenza e antipneumocócica, há alerta para a muito provável subnotificação e a falta de assistência para os casos de infarto agudo do miocárdio durante a pandemia da COVID-19.^21 , 22^ Nesse contexto, a criação de rotas e fluxos voltados para dar atenção a esses pacientes precisa de ampla estruturação e divulgação. As Figuras 1 e 2 apresentam sugestões de protocolos assistenciais que precisam ser validados e ajustados às diferentes realidades locais. Para tanto, equipamentos de proteção individual (óculos antirrespingos, protetores faciais, máscaras N95 ou equivalentes, gorros e aventais impermeáveis) para paramentação completa devem estar disponíveis para toda a equipe assistencial e seguir rígidas rotinas institucionais no seu uso.

**Figura 2:**
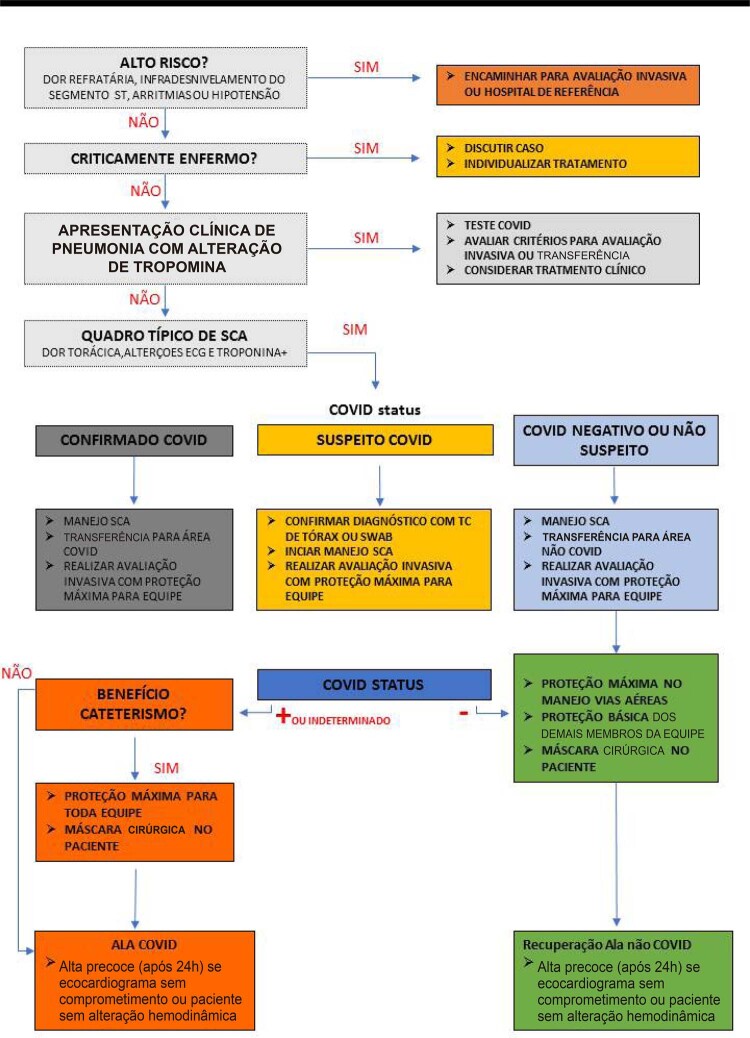
– *Angina instável/síndrome coronariana sem supra ST na era COVID.*

Somado a isso, a criação de redes de infarto, apoiadas por telemedicina, pode diminuir a mortalidade e o tempo de hospitalização. O programa Mission Lifeline STEMI Systems Accelerator^23^ observou o impacto da implementação de redes de infarto em 167 hospitais, que atenderam a 23.498 pacientes com infarto com supradesnivelamento do segmento ST. Documentou-se melhora de 3 processos-chave para o cuidado: ativação pré-hospitalar da hemodinâmica (62% para 91%; p < 0,001); protocolo de chamada única para transferência de unidade externa (45% a 70%; p < 0,001); encaminhamento direto para a hemodinâmica (evitando-se atrasos no pronto-socorro) (48% a 59%; p = 0,002); além de significativa redução do tempo entre o primeiro contato médico até o balão (88 minutos × 98 minutos; p < 0,001). O programa LATIN^24 - 28^ conectou 13 hospitais terciários a 86 unidades de pronto atendimento (UPA) no Brasil. Mais de 6.000 pacientes com dor torácica foram atendidos através de telemedicina. O tempo médio para o diagnóstico de infarto foi de 5 minutos. A angioplastia primária foi empregada em 49% desses pacientes, atingindo-se mortalidade hospitalar média de 5%. Nessas redes, casos atendidos precocemente seguem rotas que evitam o pronto atendimento e conduzem o paciente diretamente à sala de hemodinâmica, encurtando os retardos evitáveis, e podem até prescindir de UTI, aliviando a sobrecarga ao sistema de saúde.

### Perspectivas Futuras

A inerente e iminente recessão econômica dificulta a manutenção da população em quarentena. Fato que, teoricamente, poderá implicar na maior disseminação da doença ou surgimento de uma segunda onda com chances reais de superlotação e esgotamento no sistema de saúde. Nesse sentido, compilar ambiente seguro e protocolos adequados de tratamento dos pacientes com SCA é fundamental no enfrentamento da pandemia, tanto no âmbito da saúde pública como suplementar. Revisão contínua das medidas institucionais de gerenciamento protocolar são fundamentais no manejo dos pacientes com COVID-19 que apresentam SCA e para aqueles com doença arterial coronariana aguda sem infecção coexistente. A equipe médica deverá estar sempre alinhada e trabalhando de forma multidisciplinar, atenta aos potenciais efeitos colaterais cardiológicos das diferentes medicações e terapêuticas utilizadas no enfrentamento da COVID-19. Será imperativo o treinamento da equipe de atendimento com relação a: triagem, biossegurança, escalas de trabalho, equipamentos de proteção individual, técnicas corretas de paramentação, observância absoluta nos processos de desparamentação, cuidados com pacientes, isolamento, medidas de higienização, adequação diagnóstica e terapêutica, de modo a exposição da equipe de saúde. Aliado a todo esse preparo, é urgente alertar a população de que “o infarto e as doenças do coração não respeitam quarentena”. Campanhas dedicadas, como a *Coração Alerta* (https://coracaoalerta.com.br), patrocinada pela Sociedade Brasileira de Hemodinâmica e Cardiologia Intervencionista (SBHCI), ações governamentais, sociais e comunitárias e espaços para este fim na mídia leiga e literatura médica podem, como nunca, salvar vidas.

Um novo modo de viver e de fazer assistência se apresentou e segue em nosso horizonte. O real desfecho de tudo o que estamos vivenciando ainda não é sabido, mas é certo que esta complicada situação irá passar, e as patologias cardiovasculares (em especial as SCA) não podem ser colocadas em segundo plano – o melhor manejo disponível sempre deverá estar disponível e ser oferecido. Com ciência, sabedoria e bom senso, sairemos desta grave situação, mais fortes e com muitos ensinamentos, o que nos ajudará ainda mais a qualificar a atividade assistencial em prol de nosso bem maior: a vida.
