# Supplementary material for: Síndromes Coronarianas Agudas no Contexto Atual da Pandemia COVID-19
Source: Arq Bras Cardiol. 2020 Jun 29;114(6):1067–71. [Article in Portuguese] doi: 10.36660/abc.20200358 (PMC8416128; doi:10.36660/abc.20200358)
Supplement: Translation

# Translation: Acute Coronary Syndromes in the Current Context of the Covid-19 Pandemic

**Authors:** Raphael Boesche Guimarães, Breno Falcão, Ricardo Alves Costa, Marcelo Antônio Cartaxo Queiroga Lopes, Roberto Vieira Botelho, Ricardo Petraco, Rogério Sarmento-Leite

**Affiliations:** 1 Brazil Instituto de Cardiologia, Porto Alegre, RS - Brazil; 2 Brazil Hospital de Messejana, Fortaleza, CE - Brazil; 3 Brazil Hospital Universitário Walter Cantídeo, Fortaleza, CE - Brazil; 4 Brazil Instituto Dante Pazzanese de Cardiologia, São Paulo, SP - Brazil; 5 Brazil Hospital Sírio-Libanês, São Paulo, SP - Brazil; 6 Brazil Hospital Alberto Urquiza Wanderley, João Pessoa, PB - Brazil; 7 Brazil Instituto do Coração do Triângulo, Uberlândia, MG - Brazil; 8 Reino Unido Imperial College London, Londres, Inglaterra Reino Unido; 9 Brazil Hospital Moinhos de Vento Porto Alegre, RS - Brazil; 10 Brazil Universidade Federal de Ciências da Saúde de Porto Alegre, Porto Alegre, RS - Brazil

**Keywords:** Acute Coronary Syndrome/complications, Coronavirus, COVID-19, Pandemics, Acute Myocardial Infarction/prevention and control, Telemedicine/trends, Quarantine

## Introduction

COVID-19, initially described at the end of 2019 in China, may present as atypical pneumonia and severe respiratory failure.^1^ Classified in February 2020 as a pandemic^2^ by the World Health Organization (WHO), COVID-19 has had major clinical, social, political and economic implications. Society as a whole has had to adapt to a new reality, forcing hospitals to rewrite their routine practices and clinical pathways. Specific measures have had to be put in place to prevent hospital transmission of the infection. Also, dedicated COVID units have been set up and infection control protocols implemented. Finally, additional human, material and financial resources have been allocated in order to provide patients with the best possible care, without compromising the safety of healthcare workers.

Social isolation as a way of containing the spread of the disease may have helped, in some places, to “flatten the curve”, preventing the collapse of healthcare systems. However, the duration of the pandemic, as well as the precise risk of transmission are still largely unknown.

Typical symptoms of COVID-have been described^3 , 4^ and most infected patients present with mild viral syndromes. As a result, and as part of social distancing measures, patients are recommended to seek hospital care only in case of severe symptoms. This policy has, on the other hand, has generated a widespread reluctance by the population to go to hospitals, for fear of being exposed to the virus in healthcare settings. As a result, diagnosis, treatment and prognosis of several other clinical conditions have been unintentionally impacted. This applies to cardiology and, particularly, to Acute Coronary Syndromes (ACS), a phenomenon described globally.^5^

Furthermore, individuals who are over 60 years old and those with previous cardiovascular or respiratory disorders are more likely to develop severe forms of COVID-19, with increased cardiovascular compromise during the infection course, such as myocarditis, type II infarctions and thromboembolic phenomena.^6 , 7^

### International Experiences

Experiences shared from countries in which the COVID-19 infection wave preceded ours indicate important associations between COVID-19 and cardiovascular disease.

COVID-19 patients with established cardiovascular disease and those with hypertension and diabetes represent about 40% of the severe cases and have a worse prognosis.^8^ These groups have a much higher fatality rate – 7.3-10.5% compared to 2.3% for the general population^9^ . Cardiac manifestations attributed to COVID-19 have also been reported, with arrhythmias occurring in 16.7% and acute myocardial injury in up to 7% of hospitalized cases.^10 , 11^

In addition to these direct associations, the “side effects” of the COVID-19 pandemic in the care of acute coronary syndromes have generated concern. There was a sharp drop in the search for cardiac emergency room care by patients with ACS, possibly related to the fear of contracting infections in the hospital environment, which can result in underdiagnosis and inadequate treatment, with risk of death and long-term morbidity.^12 , 13^ In addition, delays in primary angioplasty have been reported, with complications of late-presenting myocardial infarction having been described.^14^

National campaigns targeted to raise awareness of ACS symptoms have been put in place internationally, advising patients to seek help quickly in case of suspected cardiac emergencies.^15^ Telemedicine is a facilitating tool for such complex COVID-19 circumstances, as it has the potential to allow the physician to remotely recognize suspected symptoms of acute coronary syndrome and guide the patient to seek care immediately. In addition, it allows pre-hospital diagnosis of acute ST-elevation myocardial infarction, enabling the rapid activation of catheterization laboratories and selection of the best myocardial reperfusion strategy using an individualized approach. It is possible to refer the patient directly to the heart attack center or catheterization laboratory, by-passing emergency departments, reducing hospitalization time and myocardial sequelae.^16 , 17^

Therefore, early training of cardiology teams with pathways that include the use of telemedicine is essential for the successful implementation of such tools.

### Management of ACS *(protocols of care)*

The COVID-19 pandemic has impacted the management of acute coronary syndromes,^18^ particularly the speed at which proven medical and interventional therapies can be implemented.^19^ For instance, in STEMI cases, reperfusion is known to be most beneficial if implemented within the first few hours of symptom onset.^20^ With early actions, there is a reduction of ventricular arrhythmias, reduced myocardial damage, lower incidences of reinfarction and greater preservation of ventricular function.^21^ Unfortunately, late presentations of ACS have been reported worldwide. In New York, United States, there has been a reduction of up to 70% in the volume of emergency calls due to ACS and an increase of up to 800% in sudden deaths.^22 , 23^

Recommendations from several Medical Societies^19 , 20^ highlight the cardiovascular clinical implications of coronavirus and call for attention to be paid to individual and populational risks.^20 , 21^ In addition to public health strategies to prevent the spread of infection, such as influenza and pneumococcal vaccination, there is a warning of a very likely underreporting and lack of assistance for cases of acute myocardial infarction during the COVID-19 pandemic.^21 , 22^ In this context, the creation of routes and flows aimed at the care of these patients need extensive structuring and dissemination. Figures 1 and 2 depict suggestions of care pathways that can be adjusted to local hospital circumstances. For this purpose, personal protective equipment (anti-splash goggles, facial protectors, respirator masks, caps and waterproof aprons/gowns) should be available to the entire team with strict institutional routines for their use.

**Figure 1:** – *Acute ST elevation myocardial infarction in the COVID era.*

**Figure 2:** – *Unstable Angina/Coronary Syndrome Without ST elevation in the COVID era.*

In addition, the creation of infarction networks, supported by telemedicine, can reduce mortality and length of hospitalization. The “Mission: Lifeline STEMI Systems Accelerator”^23^ program observed the impact of the implementation of infarction networks in 167 hospitals, which treated 23,498 patients with acute ST elevation myocardial infarction. It documented key processes for improved care: pre-hospital catheterization laboratory activation (62% to 91%; P<0.001), single call protocol for external unit transfer (45% - 70%; P<0.001), and direct referral to the laboratory (avoiding delays in the emergency room) (48%- 59%; P=0.002). There was also a significant reduction in the time between the first medical contact and balloon inflation (88 minutes x 98 minutes; p<0.001). The LATIN^24 - 28^ program connected 13 tertiary hospitals to 86 emergency care units (UPAs) in Brazil. It treated more than 6,000 patients with chest pain through telemedicine. The mean time for the diagnosis of infarction was 5 minutes. Primary percutaneous coronary intervention was used in 49% of these patients, reaching an average hospital mortality of 5%. In these networks, cases assisted early follow routes that avoid emergency care and lead the patient directly to the hemodynamics room, shortening the avoidable delays, which may even prevent the need for ICU, relieving the overload of the health care system.

### Future Perspectives

The imminent economic recession caused by COVID-19 makes it challenging to maintain the population lockdown for long. This fact may theoretically imply a greater spread of the disease or the emergence of a second wave, with real chances of overcrowding and exhausting the health system. In this sense, providing a safe environment and adequate protocols for the treatment of patients with ACS is fundamental for coping with the pandemic, both in the public and supplementary health areas. The continuous review of institutional protocol management measures are fundamental for the management of patients with COVID-19 who have ACS, as well as for those without the co-existing infection. The medical staff should always be aligned and work as multidisciplinary teams, always alert to the potential cardiac side effects of the different drugs and therapies used to treat COVID-19. Training of the care team in relation to screening, biosafety, work routes, personal protective equipment, correct donning techniques, stringent observance of the doffing processes, patient care, isolation, hygiene measures, diagnostic adequacy and therapy avoiding the exposure of the health team will be imperative. Combined with all this preparation, it is urgent to warn the population that “myocardial infarction and heart diseases do not respect the quarantine”. Dedicated campaigns such as *Coração Alerta* (https://coracaoalerta.com.br) sponsored by the Brazilian Society of Hemodynamics and Interventional Cardiology (SBHCI), governmental, social and community actions and spaces for this purpose in the lay media and medical literature, as never before, can save lives.

A new way of living and providing care has emerged. The real final outcome of everything we are experiencing is not yet known, but what is certain is that this acute complicated situation will pass and cardiovascular pathologies, especially ACS, cannot be put in the background. Therefore, the best available management should always be available and offered. With science, wisdom and common sense we will come out stronger out of this serious situation with many teachings that will further help us to qualify our care activity for the greater good, which is the protection of life.
